# Distinct Clinical Characteristics in Young-Onset Pancreatic Neuroendocrine Tumor

**DOI:** 10.3390/cancers12092501

**Published:** 2020-09-03

**Authors:** Suleyman Yasin Goksu, Muhammet Ozer, Syed Mohammad Ali Kazmi, Nina Niu Sanford, Todd A. Aguilera, Chul Ahn, David Hsiehchen, Aravind Sanjeevaiah, Leticia Khosama, Jonathan Bleeker, Muslim Atiq, Muhammad Shaalan Beg

**Affiliations:** 1 Division of Hematology and Oncology, UT Southwestern Medical Center, Dallas, TX 75390, USA; suleyman.goksu@utsouthwestern.edu (S.Y.G.); MOzer@capitalhealth.org (M.O.); Syed.Kazmi@utsouthwestern.edu (S.M.A.K.); david.hsieh@utsouthwestern.edu (D.H.); aravind.sanjeevaiah@utsouthwestern.edu (A.S.); Leticia.Khosama@utsouthwestern.edu (L.K.); 2 Department of Internal Medicine, UT Southwestern Medical Center, Dallas, TX 75390, USA; 3 Department of Internal Medicine, Capital Health Regional Medical Center, Trenton, NJ 08638, USA; 4 Department of Radiation Oncology, UT Southwestern Medical Center, Dallas, TX 75390, USA; Nina.Sanford@UTSouthwestern.edu (N.N.S.); Todd.Aguilera@UTSouthwestern.edu (T.A.A.); 5 Department of Population and Data Sciences, UT Southwestern Medical Center, Dallas, TX 75390, USA; chul.ahn@utsouthwestern.edu; 6 Division of Hematology and Oncology, Sanford Medical Center, Sioux Falls, SD 57117, USA; Jonathan.Bleeker@sanfordhealth.org; 7 Division of Gastroenterology and Hepatology, Sanford Medical Center, Sioux Falls, SD 57117, USA; muslim.atiq@sanfordhealth.org

**Keywords:** neuroendocrine tumors, pancreas, database, young adult, age of onset, multiple endocrine neoplasia, mutation, propensity score

## Abstract

**Simple Summary:**

The impact of age and socioeconomic factors on the outcomes of patients with pancreatic neuroendocrine tumors is understudied. In this study, we investigated the association of clinical and genomic characteristics on the survival of young- versus typical-onset pancreatic neuroendocrine tumors. We used a large national dataset and reported that patients with young-onset pancreatic neuroendocrine tumors who underwent surgery represent a disease with distinct clinical features and improved survival. Younger patients also had a lower rate of multiple endocrine neoplasia type-1 (MEN-1) mutation, which is associated with multiple microtumors and unfavorable outcomes. Understanding these differences between patients with young- versus typical-onset pancreatic neuroendocrine tumors can improve our ability to address the effect of these factors on cancer outcomes.

**Abstract:**

Background: We aimed to study the effect of socioeconomic differences and molecular characteristics on survival in patients with young-onset pancreatic neuroendocrine tumors (YOPNET) and typical-onset PNET (TOPNET). Methods: We identified the patients with YOPNET (<50 years) and TOPNET (≥50 years) who underwent definitive surgery diagnosed between 2004 and 2016 using the National Cancer Database. We evaluated overall survival (OS) using the Kaplan–Meier and Cox regression methods before and after propensity score matching. A publicly available genomic dataset was used to compare mutation frequencies among the two groups. Results: A total of 6259 patients with PNET were included, of which 27% were YOPNET. Patients with YOPNET were more likely to be Black, Hispanic, female, and have private insurance versus patients with TOPNET (all *p* < 0.001). Patients with YOPNET had a lower comorbidity score, but higher stage and tumor size (all *p* < 0.001). YOPNET was associated with a greater improved OS than TOPNET before and after propensity score matching (*p* < 0.001). On multivariable analysis, this survival difference persisted for YOPNET as an independent prognostic factor (unmatched *p* = 0.008; matched *p* = 0.01). For genomic analysis, patients with YOPNET had a lower rate of multiple endocrine neoplasia type-1 (MEN-1) mutation than patients with TOPNET (26% vs. 56%, *p* < 0.001). Conclusions: YOPNET represents a disease with distinct clinical features. Patients with YOPNET who underwent definitive surgery had better OS than patients with TOPNET despite having higher stage and tumor size. YOPNET also had lower rate of MEN-1 mutation.

## 1. Introduction

Pancreatic neuroendocrine tumors (PNETs) originate from the islet cells of the pancreas and account for 1–2% of primary pancreatic neoplasms [1]. The incidence and prevalence of PNETs are increasing in the general population due to the indolent course of the disease, ongoing improvements in imaging modalities, aging population, and increased awareness of the diagnosis [2].

The effect of age and sociodemographic differences on the outcome of patients with PNETs is understudied. The average age of diagnosis of PNET is 58, and those diagnosed before the age of 50 have been classified as young-onset pancreatic neuroendocrine tumors (YOPNET) [3]. These tumors may represent a disease group with distinct clinical and molecular features [3,4,5]. PNETs can be sporadic or familial genetic syndromes, including multiple endocrine neoplasia-1 (MEN-1), von-Hippel Lindau (VHL), neurofibromatosis-1, tuberous sclerosis (TS). In this report, we aimed to study the association of genetic and clinical characteristics to understand better the effect of age of diagnosis on YOPNET pathogenesis and potential management.

## 2. Results

### 2.1. Baseline Characteristics

We selected a total of 6259 patients with PNET who underwent definitive surgery; 1692 (27%) had YOPNET, while 4567 (73%) had typical-onset PNET (TOPNET). The median age was 42 years for YOPNET and 62 years for TOPNET. Patients with YOPNET were more likely to be female (55% vs. 47%), non-Hispanic Black (16% vs. 11%), and having private insurance (78% vs. 50%) compared to patients with TOPNET (all *p* < 0.001). Patients with YOPNET were likely to have less comorbidity score (81% vs. 68%) but greater tumor size (>4 cm) (32% vs. 27%) and higher the tumor, node, metastasis (TNM) stage (15% vs. 11%) than patients with TOPNET (all *p* < 0.001). Patients with YOPNET were less likely to be treated in an academic center versus patients with TOPNET (45% vs. 65%, *p* < 0.001). Patients with YOPNET were more likely to have a tumor in the head of the pancreas (29% vs. 27%, *p* = 0.004). In addition, patients with YOPNET were more likely to travel a longer distance to the treatment center as compared to patients with TOPNET (28% vs. 26%, *p* = 0.038). Income, education level, rurality, grade, nodal status was similar among groups. After the propensity score matching, the following variables were significantly different: race/ethnicity, comorbidity score, facility type, facility location, insurance status, and income (Table 1).

### 2.2. Survival Analyses

**Figure 1:**
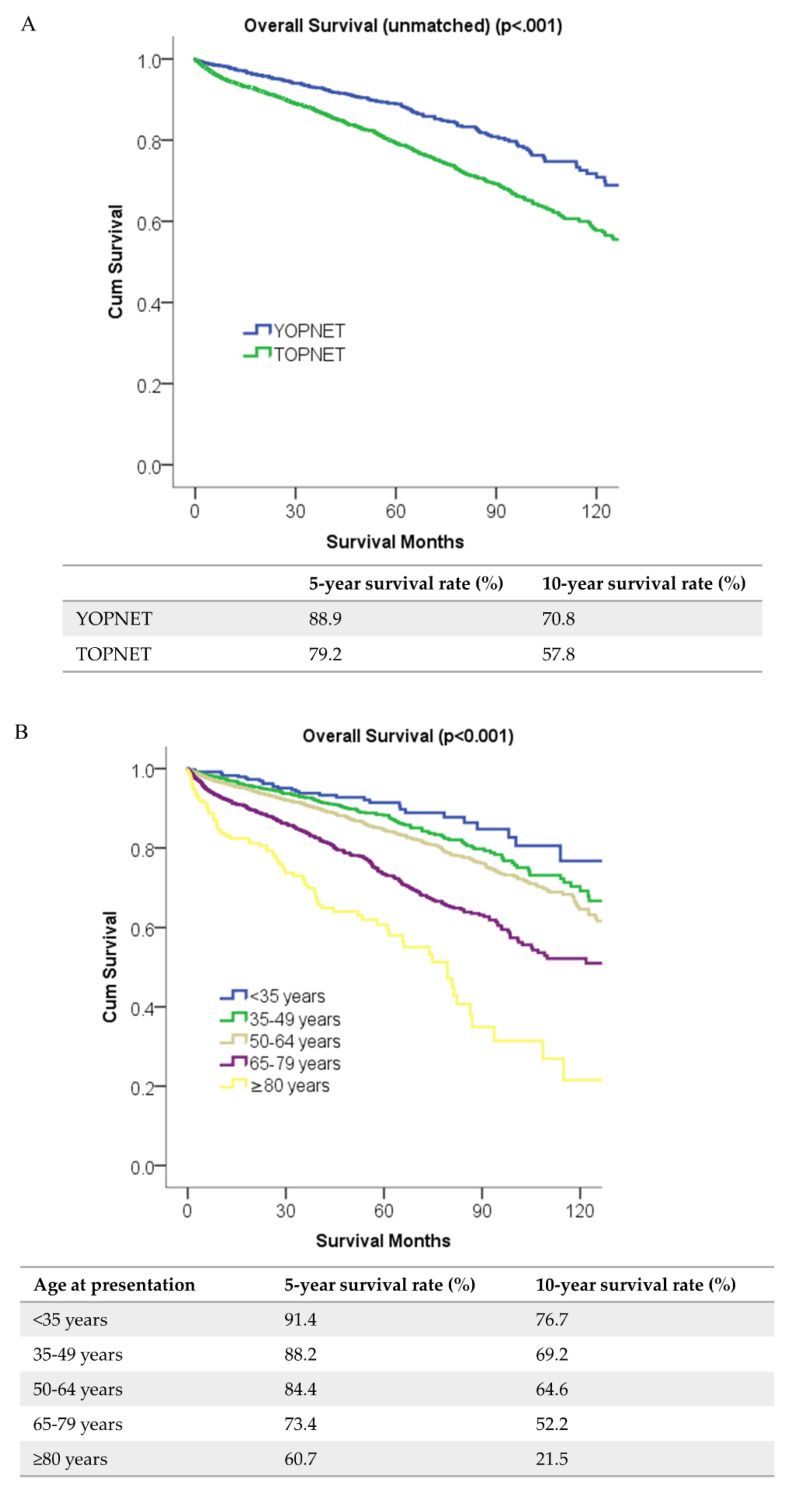
(**A**) Overall survival for unmatched groups, (**B**) the trends of survival in pancreatic neuroendocrine tumors with increasing age (YOPNET: Young-onset pancreatic neuroendocrine tumors, TOPNET: Typical-onset pancreatic neuroendocrine tumors).

Patients with YOPNET had better survival compared with patients with TOPNET before and after propensity score matching (5-year survival rate unmatched 89% vs. 79%, *p* < 0.001; matched 89% vs. 81%, *p* < 0.001) (Figure 1A). Ten-year survival rate was 71% for patients with YOPNET, 58% for patients with TOPNET for unmatched population. This association was confirmed with age groups (<35, 35–49, 50–64, 65–79, ≥80), and overall survival (OS) lowered with increasing age at presentation (Figure 1B). We used multivariable Cox regression method to identify if YOPNET was an independent factor of OS after adjusting for confounding factors. After multivariable Cox regression analysis, patients with YOPNET had better OS compared to patients with TOPNET (Hazard ratio [HR] 0.77 (1.09–1.13), *p* = 0.008) (Table 2). This difference persisted after propensity score matching (HR 0.74 (0.59–0.93), *p* = 0.01). In addition, several socioeconomic and demographic factors were independent predictors of better OS: female, comorbidity score “0”, government insurance, location of body/tail, smaller tumor size (<2 cm), negative lymph node, lower stage (I-II), lower grade, receiving chemotherapy (Table 2).

### 2.3. Genomic Analysis

We selected a total of 177 patients with PNET; 48 (27%) had YOPNET, 129 (73%) had TOPNET. The most common mutations seen in both groups were MEN-1, death-domain-associated protein (DAXX), tuberous sclerosis 2 (TSC2) (Table 3). Patients with YOPNET were less likely to have MEN-1 mutation compared to patients with TOPNET (26% vs. 56%, *p* < 0.001). This difference persisted after the Benjamini–Hochberg correction (*p* = 0.04). Patients with YOPNET had a higher rate of TSC2 mutation (24% vs. 16%, *p* = not significant (NS)), whereas patients with TOPNET had a higher rate of DAXX mutation (36% vs. 26%, *p* = NS). Other mutations were also similar among groups (Table 3).

## 3. Discussion

In our study of a national database, patients with YOPNET had better OS compared to patients with TOPNET. This difference persisted after adjusting for observable characteristics, and the propensity score matched. This improved survival was observed despite patients with YOPNET having greater tumor size and higher stage.

A single center study of 190 PNET patients revealed 33.1% with YOPNET (<50 years), and this was associated with better survival [3]. Patients with YOPNET were more likely diagnosed at an advanced stage, and the tumor was in the head of the pancreas. In contrast, in other solid malignancies, including colorectal, prostate, breast and gastric neoplasms, younger age has been associated with poor prognosis [6,7,8,9].

Previous studies have suggested that young age is associated with better OS in patients with PNET. Halfdanarson et al. studied temporal trends of PNETs, as well as changes in incidence and prognostic factors, using the Surveillance, Epidemiology, and End Results (SEER) data. In unadjusted and multivariable analysis, they reported that older age at diagnosis was associated with poor survival [1]. Median OS in patients who were <50 years was 55 months, while median OS in patients between 51 and 60 years and 61 and 70 years was 44 and 19 months, respectively. A previous study using the National Cancer Database (NCDB) data for patients with resected PNET diagnosed between 1985 and 2004 presented a prognostic model. They suggested that older age was significantly associated with an increased risk of death. Compared with patients younger than 55 years, 55- to 75-years old had a hazard ratio of 1.57 (95% CI 1.28–1.91), and those older than 75 years had a hazard ratio of 3.04 (95% CI 2.17–4.25) [10]. Due to newer imaging modalities, rising incidence of pancreatic neuroendocrine tumor (PNET), and new treatment options, we sought to build on these prior reports by evaluating the effect of young age on the outcome of PNET in the modern era. We also employed propensity score matching and adjusted for the observable difference between the two groups.

Other prognostic factors were identified from previous studies including, histologic grade, stage, tumor size, surgical treatment strategies, and germline mutation status [11,12]. In our study, patients with YOPNET were more likely diagnosed at an advanced stage, and the tumor was in the head of the pancreas. Liu et al. reported that the 5-year survival rate for stage I tumors ranges from 90% to 100%, while patients with stage IV disease have a 5-year survival rate of up to 60% [13]. The grade of PNET is a well-established predictor of survival; there was no difference in tumor grade between YOPNET and TOPNET [14].

We evaluated the effect of social and demographic differences on the outcome of YOPNET. In our study, patients with YOPNET were more likely to be female and Black (all *p* < 0.001). Other studies have suggested a male predominance reported in PNETs, and this was related to worse survival outcomes [1,15,16]. Patients with YOPNET were more likely to have private insurance, which may be a factor of older patients having Medicare coverage. We did not see any differences in socioeconomic factors, including income, education level, and distance from the medical facility. This study was limited to patients who underwent surgery and therefore represented a skewed sample.

In the genomic analysis, patients with YOPNET had a lower rate of MEN-1 mutation than patients with TOPNET. Other mutations were not different between the two groups. Positive family history and germline mutation status are significantly associated with poor survival. The majority of PNETs are sporadic, but some are associated with genetic syndromes, such as multiple endocrine neoplasia type 1 (MEN-1), von Hippel-Lindau (VHL) disease, neurofibromatosis type 1 (NF-1), and tuberous sclerosis (TS) [17]. MEN-1 is a rare autosomal dominant endocrine tumor syndrome characterized by the combination of tumors in multiple endocrine organs [18]. Nearly 50% of MEN-1 patients have multiple PNETs [19]. MEN-1 related PNETs tend to be multifocal pancreatic microadenomas, which may be responsible for worse survival outcomes [20,21]. In a study with MEN-1 patients, Nell et al. reported the median age of the patients as 41 [22]. MEN-1 related PTENs tend to be more aggressive and multifocal [21]. Likely, a higher rate of MEN-1 mutation contributes to the unfavorable prognosis of TOPNET. PNET is detected in 12–17% of patients with VHL, and 1.8% in TS [23,24,25].

We evaluated a large cohort and evaluated the effect of age at diagnosis on survival and reported on genomic differences between the two groups. This study is limited due to using predefined variables of an extensive database. NCDB does not report the cancer-specific cause of death. In a study of patients with PNET who tend to be young and have a fairly indolent disease course, the cause of death can provide valuable information. Patients with TOPNET had a higher comorbidity score, which can adversely affect survival; however, the difference in survival persisted after propensity score matching, suggesting that the age group is an independent driver of survival. NCDB does not have information regarding symptom burden, laboratory values, and clinical presentation. We were not able to assess the effect of some known prognostic markers such as Ki-67 index, performance status, and other known pathologic features such as immunohistochemistry for chromogranin. In addition, NCDB does not capturedata on disease recurrence and details on subsequent therapies.

## 4. Materials and Methods

We extracted the data using the National Cancer Database. The National Cancer Database is one of the largest databases in the U.S. and covers ~70% of cancer cases, including patient demographics, socioeconomic status, and tumor characteristics (https://www.facs.org/quality-programs/cancer/ncdb). This study was deemed exempt from the institutional review board.

### 4.1. Study Population

**Figure 2:**
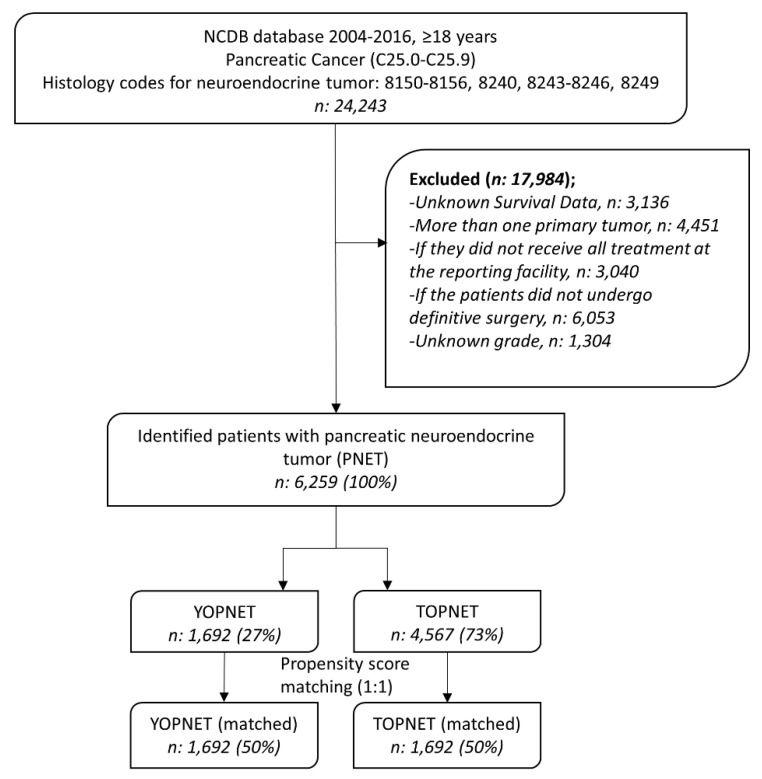
A diagram for inclusion and exclusion criteria. (YOPNET: young-onset PNET, TOPNET: typical-onset PNET).

We identified 24,243 adult patients (≥18 years) with pancreatic neuroendocrine tumors diagnosed between 2004 and 2016. We used the “C25.0–C25.9” ICD-O-3/WHO 2008 site recode and “8150–8156, 8240, 8243–8246, 8249” ICD-O-3 histologic codes to select patients with pancreatic neuroendocrine tumor [2,26,27,28]. We included patients with PNET who underwent definitive surgery, which is defined using “30–80” surgery codes, excluding no surgery, local excision, surgery not specified, and unknown surgery [29]. We excluded 17,984 patients who had unknown survival data, more than one primary tumor, were not receiving all treatments at the reporting facility, not undergoing definitive surgery, and unknown grade (Figure 2).

### 4.2. Primary Interest

We divided the patient population into two groups based on the age of diagnosis. Those diagnosed with PNET at the age of less than 50 years were classified as YOPNET, and those 50 years or above were classified as typical-onset PNET (TOPNET) [3,30,31,32]. In addition, the age at presentation was categorized as five groups <35, 35–49, 50–64, 65–79, ≥80 to assess the survival trends.

### 4.3. Covariables

We used patient demographics (sex (male, female), race/ethnicity (non-Hispanic White, non-Hispanic Black, Hispanics, other), Charlson–Deyo Score (0–2+), facility type (academic, non-academic, other), facility location (New England, Middle Atlantic, South Atlantic, East North Central, East South Central, West North Central, West South Central, Mountain, Pacific), socioeconomic status (rurality (metropolitan, non-metropolitan), education level (rates of patients without high school level ≥21%, 13–20.9%, 7–12.9%, <7%), median income quartiles (<USD 38,000, USD 38,000–USD 47,999, USD 48,000–USD 62,999, >USD 63,000), insurance status (uninsured, private insurance, government insurance), travel distance to treatment facility (<12.5, 12.5–49.9, ≥50 miles)), tumor characteristics (primary site (head, body/tail, other), NCDB analytic stage group (I-IV), pathological grade (I-IV) [2,28], tumor size (<2, 2–4, >4 cm), nodal status (negative, positive), and treatments (radiotherapy (yes, no), and chemotherapy (yes, no)) [27,30]. Travel distance was defined using great circle distance, which calculates the distance between the patient’s residence and the reporting facility, using the geographic centroid of zip codes [33]. National Cancer Database provides the data on education level, which is determined using the zip code of the patient’s residence area based on census data and then stratified by quartiles.

NCDB analytic stage is the TNM American Joint Committee on Cancer (AJCC) pathological stage group, but it can use the TNM AJCC clinical stage group when the pathological stage is not available [34]. We stratified the stage as I-II (early), and III-IV (advanced) [30]. The Charlso–Deyo score shows comorbid conditions, which contains 19 different diseases, and each disease has a different score between 1 and 6 [35].

### 4.4. Patient Selection for Genomic Analysis

The American Association for Cancer Research Genomics Evidence Neoplasia Information Exchange (AACR GENIE) shares open access clinical and genomic datasets for precision cancer medicine research with multiple tumor types, including 10,000 patients. We identified 177 patients with pancreatic neuroendocrine tumors who had genomic data from Memorial Sloan Kettering-Integrated Mutation Profiling of Actionable Cancer Targets (MSK-IMPACT) using the AACR GENIE v7-public [36]. Unknown age was excluded. As aforementioned above, we categorized age groups as <50 years (YOPNET), and ≥50 years (TOPNET). We evaluated 319 genes, which were included in MSK-IMPACT, and compared mutation frequencies between patients with YOPNET and TOPNET.

### 4.5. Statistical Analysis

We used the chi-square test or Fisher’s exact test to compare patients with YOPNET and TOPNET for categorical variables. We performed the Kaplan–Meier method with a log-rank test for univariable overall survival analysis. Patients who were alive at the last follow-up were censored. Multivariable Cox regression method was used to identify YOPNET as an independent prognostic factor after adjusting for demographics (sex, race/ethnicity, comorbidity score, facility type), socioeconomic status (rurality, income, insurance, travel distance), tumor characteristics (primary site, grade, stage, tumor size, nodal status), chemotherapy. We provided the hazard ratio (HR) with 95% confidence interval (CI). Missing data were included in the analysis as unknown categorical variables. All tests were reported with two-sided *p*-value (*p* < 0.05 was considered statistical significance) using the SPSS version 25.0.

We performed 1:1 nearest neighbor propensity score matching to reduce the selection bias adjusting for demographics, socioeconomic status, tumor characteristics, and treatment variables using R software version 3.6.2 with MatchIt package (Nonparametric Preprocessing for Parametric Causal Inference) [37,38]. After the propensity matched, we reanalyzed overall survival using the Kaplan–Meier and Cox regression method.

For genomic analysis, we used Fisher’s exact test with Benjamini–Hochberg correction to compare mutation frequencies between YOPNET and TOPNET.

## 5. Conclusions

Patients with YOPNET who underwent surgery had a better OS than TOPNET despite having a higher stage and greater tumor size. Tumor grade was not different between the two groups. Patients with YOPNET had a lower rate of MEN-1 germline mutation, which could provide a molecular reason for better survival in that group.

## Figures and Tables

**Table 1:** Baseline characteristics of patients with YOPNET vs. TOPNET.

Characteristics	Before Propensity Score Matching		*p-Value*	After Propensity Score Matching		*p-Value*
	YOPNET (%)	TOPNET (%)		YOPNET (%)	TOPNET (%)	
	1692 (27.0)	4567 (73.0)		1692 (50.0)	1692 (50.0)	
**Age at diagnosis (median)**	42	62		42	60	
**Sex**			<0.001			NS
**Male**	769 (45.4)	2412 (52.8)		769 (45.4)	822 (48.6)	
**Female**	923 (54.6)	2155 (47.2)		923 (54.6)	870 (51.4)	
**Race/Ethnicity**			<0.001			<0.001
**Non-Hispanic White**	1122 (66.3)	3493 (76.5)		1122 (66.3)	1218 (72.0)	
**Non-Hispanic Black**	266 (15.7)	499 (10.9)		266 (15.7)	183 (10.8)	
**Hispanics**	142 (8.4)	217 (4.8)		142 (8.4)	97 (5.7)	
**Other/Unknown**	162 (9.6)	358 (7.8)		162 (9.6)	194 (11.5)	
**Year of diagnosis**			0.004			NS
**2004–2006**	145 (8.6)	376 (8.2)		145 (8.6)	156 (9.2)	
**2007–2009**	317 (18.7)	692 (15.2)		317 (18.7)	292 (17.3)	
**2010–2012**	474 (28.0)	1295 (28.4)		474 (28.0)	473 (28.0)	
**2013–2015**	756 (44.7)	2204 (48.3)		756 (44.7)	771 (45.6)	
**Comorbidity Score**			<0.001			0.03
**0**	1377 (81.4)	3100 (67.9)		1377 (81.4)	1324 (78.3)	
**1**	260 (15.4)	1116 (24.4)		260 (15.4)	317 (18.7)	
**2+**	55 (3.3)	351 (7.7)		55 (3.3)	51 (3.0)	
**Facility Type**			<0.001			<0.001
**Academic**	765 (45.2)	2986 (65.4)		765 (45.2)	777 (45.9)	
**Non-academic**	288 (17.0)	1581 (34.6)		288 (17.0)	915 (54.1)	
**Others**	639 (37.8)	0 (0)		639 (37.8)	0 (0)	
**Facility Location**			<0.001			<0.001
**New England**	40 (2.4)	183 (4.0)		40 (2.4)	37 (2.2)	
**Middle Atlantic**	185 (10.9)	836 (18.3)		185 (10.9)	189 (11.2)	
**South Atlantic**	264 (15.6)	1016 (22.2)		264 (15.6)	309 (18.3)	
**East North Central**	158 (9.3)	781 (17.1)		158 (9.3)	246 (14.5)	
**East South Central**	64 (3.8)	287 (6.3)		64 (3.8)	124 (7.3)	
**West North Central**	107 (6.3)	435 (9.5)		107 (6.3)	187 (11.1)	
**West South Central**	78 (4.6)	377 (8.3)		78 (4.6)	172 (10.2)	
**Mountain**	42 (2.5)	181 (4.0)		42 (2.5)	112 (6.6)	
**Pacific**	115 (6.8)	471 (10.3)		115 (6.8)	316 (18.7)	
**Unknown**	639 (37.8)	0 (0)		639 (37.8)	0 (0)	
**Insurance status**			<0.001			<0.001
**Uninsured**	78 (4.6)	89 (1.9)		78 (4.6)	82 (4.8)	
**Private**	1314 (77.7)	2273 (49.8)		1314 (77.7)	1215 (71.8)	
**Government**	267 (15.8)	2120 (46.4)		267 (15.8)	382 (22.6)	
**Unknown**	33 (2.0)	85 (1.9)		33 (2.0)	13 (0.8)	
**Income**			NS			0.009
**<USD 38,000**	270 (16.0)	683 (15.0)		270 (16.0)	236 (13.9)	
**USD 38,000–USD 47,999**	345 (20.4)	965 (21.1)		345 (20.4)	339 (20.0)	
**USD 48,000–USD 62,999**	409 (24.2)	1230 (26.9)		409 (24.2)	485 (28.7)	
**>USD 63,000**	664 (39.2)	1672 (36.6)		664 (39.2)	621 (36.7)	
**Unknown**	4 (0.2)	17 (0.4)		4 (0.2)	11 (0.7)	
**Education**			NS			NS
**≥21%**	269 (15.9)	651 (14.3)		269 (15.9)	245 (14.5)	
**13–20.9%**	418 (24.7)	1117 (24.5)		418 (24.7)	408 (24.1)	
**7–12.9%**	521 (30.8)	1520 (33.3)		521 (30.8)	552 (32.6)	
**<7%**	481 (28.4)	1264 (27.7)		481 (28.4)	476 (28.1)	
**Unknown**	3 (0.2)	15 (0.3)		3 (0.2)	11 (0.7)	
**Rurality**			NS			NS
**Metropolitan**	1430 (84.5)	3757 (82.3)		1430 (84.5)	1425 (84.2)	
**Non-metropolitan**	223 (13.2)	693 (15.2)		223 (13.2)	238 (14.1)	
**Unknown**	39 (2.3)	117 (2.6)		39 (2.3)	29 (1.7)	
**Travel distance**			0.038			NS
**<12.5 miles**	562 (33.2)	1690 (37.0)		562 (33.2)	597 (35.3)	
**12.5–49.9 miles**	657 (38.8)	1667 (36.5)		657 (38.8)	622 (36.8)	
**≥50 miles**	470 (27.8)	1197 (26.2)		470 (27.8)	463 (27.4)	
**Unknown**	3 (0.2)	13 (0.3)		3 (0.2)	10 (0.6)	
**Tumor location**			0.004			NS
**Head**	487 (28.8)	1214 (26.6)		487 (28.8)	437 (25.8)	
**Body/Tail**	886 (52.4)	2603 (57.0)		886 (52.4)	950 (56.1)	
**Other**	319 (18.9)	750 (16.4)		319 (18.9)	305 (18.0)	
**Tumor size (cm)**			<0.001			NS
**<2**	453 (26.8)	1466 (32.1)		453 (26.8)	473 (28.0)	
**2–4**	682 (40.3)	1819 (39.8)		682 (40.3)	677 (40.0)	
**>4**	543 (32.1)	1246 (27.3)		543 (32.1)	528 (31.2)	
**Unknown**	14 (0.8)	36 (0.8)		14 (0.8)	14 (0.8)	
**Nodal status**			0.048			NS
**Negative**	943 (55.7)	2655 (58.1)		943 (55.7)	959 (56.7)	
**Positive**	545 (32.2)	1325 (29.0)		545 (32.2)	519 (30.7)	
**Unknown**	204 (12.1)	587 (12.9)		204 (12.1)	214 (12.6)	
**Stage**			<0.001			NS
**1–2**	1239 (73.2)	3580 (78.4)		1239 (73.2)	1278 (75.5)	
**3–4**	245 (14.5)	513 (11.2)		245 (14.5)	211 (12.5)	
**Unknown**	208 (12.3)	474 (10.4)		208 (12.3)	203 (12.0)	
**Grade**			NS			NS
**I**	1290 (76.2)	3520 (77.1)		1290 (76.2)	1304 (77.1)	
**II**	329 (19.4)	808 (17.7)		329 (19.4)	307 (18.1)	
**III**	62 (3.7)	216 (4.7)		62 (3.7)	70 (4.1)	
**IV**	11 (0.7)	23 (0.5)		11 (0.7)	11 (0.7)	
**Radiotherapy**			NS			NS
**Yes**	52 (3.1)	125 (2.7)		52 (3.1)	55 (3.3)	
**No/Unknown**	1640 (96.9)	4442 (97.3)		1640 (96.9)	1637 (96.7)	
**Chemotherapy**			0.009			NS
**Yes**	120 (7.1)	242 (5.3)		120 (7.1)	124 (7.3)	
**No/Unknown**	1572 (92.9)	4325 (94.7)		1572 (92.9)	1568 (92.7)	

YOPNET: Young-onset pancreatic neuroendocrine tumors, TOPNET: Typical-onset pancreatic neuroendocrine tumors, NS: not significant.

**Table 2:** Multivariable Cox regression analysis for overall survival.

Characteristics	Unmatched		Propensity Matched	
	HR (95% CI)	*p-Value*	HR (95% CI)	*p-Value*
**Age**				
**YOPNET**	0.77 (0.63–0.93)	0.008	0.75 (0.60–0.93)	0.012
**TOPNET**	Ref		Ref	
**Sex**				
**Male**	Ref		Ref	
**Female**	0.76 (0.67–0.87)	<0.001	0.82 (0.68–0.98)	0.038
**Race/Ethnicity**				
**Non-Hispanic White**	Ref		Ref	
**Non-Hispanic Black**	0.80 (0.64–1.00)	NS	0.78 (0.56–1.08)	NS
**Hispanics**	0.88 (0.64–1.21)	NS	1.04 (0.68–1.58)	NS
**Other/Unknown**	1.03 (0.83–1.28)	NS	0.96 (0.72–1.27)	NS
**Comorbidity Score**				
**0**	Ref		Ref	
**1**	1.23 (1.06–1.42)	0.005	1.28 (1.01–1.61)	0.035
**2+**	1.73 (1.40–2.13)	<0.001	1.42 (0.89–2.29)	NS
**Facility Type**				
**Academic**	Ref		Ref	
**Non-academic**	1.12 (0.97–1.29)	NS	1.13 (0.90–1.42)	NS
**Unknown**	0.66 (0.43–1.04)	NS	0.68 (0.49–0.93)	0.017
**Travel distance**				
**<12.5 miles**	Ref		Ref	
**12.5–49.9 miles**	1.02 (0.87–1.19)	NS	1.05 (0.84–1.31)	NS
**≥50 miles**	0.84 (0.70–1.02)	NS	0.96 (0.73–1.27)	NS
**Unknown**	0.26 (0.02–2.59)	NS	0.19 (0.01–3.28)	NS
**Income**				
**<USD 38,000**	Ref		Ref	
**USD 38,000–USD 47,999**	0.88 (0.72–1.08)	NS	1.04 (0.76–1.43)	NS
**USD 48,000–USD 62,999**	0.82 (0.67–1.01)	NS	1.05 (0.78–1.41)	NS
**>USD 63,000**	0.75 (0.61–0.93)	0.01	0.90 (0.66–1.24)	NS
**Unknown**	1.72 (0.53–5.51)	NS	4.84 (0.66–35.7)	NS
**Insurance status**				
**Uninsured**	Ref		Ref	
**Private**	0.87 (0.57–1.33)	NS	0.81 (0.52–1.26)	NS
**Government**	1.65 (1.08–2.51)	0.018	1.71 (1.08–2.71)	0.02
**Unknown**	1.31 (0.73–2.36)	NS	0.77 (0.26–2.29)	NS
**Rurality**				
**Metropolitan**	Ref		Ref	
**Non-metropolitan**	1.04 (0.86–1.26)	NS	1.01 (0.75–1.36)	NS
**Unknown**	1.02 (0.68–1.53)	NS	2.24 (1.22–4.13)	0.01
**Tumor Location**				
**Head**	Ref		Ref	
**Body/Tail**	0.76 (0.66–0.88)	<0.001	0.74 (0.59–0.91)	0.005
**Other**	0.79 (0.65–0.95)	0.012	0.76 (0.58–0.99)	0.047
**Tumor size (cm)**				
**<2**	Ref		Ref	
**2–4**	1.45 (1.19–1.78)	<0.001	1.46 (1.04–2.04)	0.026
**>4**	1.71 (1.39–2.10)	<0.001	1.86 (1.33–2.61)	<0.001
**Unknown**	1.88 (1.18–3.01)	0.008	1.65 (0.77–3.53)	NS
**Nodal status**				
**Negative**	Ref		Ref	
**Positive**	1.41 (1.22–1.63)	<0.001	1.33 (1.08–1.64)	0.006
**Unknown**	0.99 (0.79–1.23)	NS	0.78 (0.54–1.14)	NS
**Stage**				
**I-II**	Ref		Ref	
**III-IV**	2.19 (1.87–2.57)	<0.001	2.39 (1.90–3.01)	<0.001
**Unknown**	1.26 (1.05–1.50)	0.011	1.38 (1.06–1.78)	0.015
**Grade**				
**I**	Ref		Ref	
**II**	1.16 (0.98–1.36)	NS	1.25 (0.99–1.59)	NS
**III**	3.09 (2.55–3.74)	<0.001	3.48 (2.63–4.59)	<0.001
**IV**	4.06 (2.62–6.30)	<0.001	6.35 (3.64–11.08)	<0.001
**Chemotherapy**				
**Yes**	Ref		Ref	
**No/Unknown**	1.67 (1.39–2.00)	<0.001	2.04 (1.61–2.59)	<0.001

YOPNET: Young-onset pancreatic neuroendocrine tumors, TOPNET: Typical-onset pancreatic neuroendocrine tumors, HR: hazard ratio, CI: confidence interval, NS: not significant.

**Table 3:** Common mutations in patients with YOPNET and TOPNET.

Genes	MSK-IMPACT			
	YOPNET (*n*/%) 48 (27%)	TOPNET (*n*/%) 129 (73%)	*p-Value*	q-Value *
**MEN1**	14 (26%)	82 (56%)	<0.001	0.04
**DAXX**	14 (26%)	52 (36%)	NS	NS
**TSC2**	13 (24%)	23 (16%)	NS	NS
**ATRX**	7 (13%)	31 (21%)	NS	NS
**TP53**	7 (13%)	21 (14%)	NS	NS
**BRAF**	5 (9%)	5 (3%)	NS	NS
**ATM**	4 (7%)	7 (5%)	NS	NS
**ARID1A**	4 (7%)	14 (9%)	NS	NS
**PTEN**	4 (7%)	12 (8%)	NS	NS
**SETD2**	3 (5%)	19 (13%)	NS	NS
**BCOR**	3 (5%)	2 (1%)	NS	NS
**TSC1**	3 (5%)	4 (3%)	NS	NS
**TERT**	3 (5%)	6 (4%)	NS	NS
**MDC1**	2 (4%)	0 (0%)	NS	NS
**RASA1**	2 (4%)	0 (0%)	NS	NS
**PPM1D**	2 (4%)	1 (1%)	NS	NS
**ATR**	2 (4%)	1 (1%)	NS	NS
**BCL6**	2 (4%)	1 (1%)	NS	NS
**INPP4B**	2 (4%)	1 (1%)	NS	NS
**KMT2D**	1 (2%)	11 (7%)	NS	NS

YOPNET: Young-onset pancreatic neuroendocrine tumors, TOPNET: Typical-onset pancreatic neuroendocrine tumors, NS: not significant, MSK-IMPACT: Memorial Sloan Kettering-Integrated Mutation Profiling of Actionable Cancer Targets. * Benjamini–Hochberg correction.

## References

[1] Halfdanarson T.R., Rabe K.G., Rubin J., Petersen G.M. (2008). Pancreatic neuroendocrine tumors (PNETs): Incidence, prognosis and recent trend toward improved survival. Ann. Oncol..

[2] Dasari A., Shen C., Halperin D., Zhao B., Zhou S., Xu Y., Shih T., Yao J.C. (2017). Trends in the incidence, prevalence, and survival outcomes in patients with neuroendocrine tumors in the United States. JAMA Oncol..

[3] Beeghly-Fadiel A., Luu H.N., Du L., Shi C., McGavic D.P., Parikh A.A., Raskin L. (2016). Early onset pancreatic malignancies: Clinical characteristics and survival associations. Int. J. Cancer.

[4] Muniraj T., Jamidar P.A., Aslanian H.R. (2013). Pancreatic cancer: A comprehensive review and update. Dis. Mon..

[5] Raimondi S., Maisonneuve P., Lowenfels A.B. (2009). Epidemiology of pancreatic cancer: An overview. Nat. Rev. Gastroenterol. Hepatol..

[6] Ballester V., Rashtak S., Boardman L. (2016). Clinical and molecular features of young-onset colorectal cancer. World J. Gastroenterol..

[7] Salinas C.A., Tsodikov A., Ishak-Howard M., Cooney K.A. (2014). Prostate cancer in young men: An important clinical entity. Nat. Rev. Urol..

[8] Gabriel C.A., Domchek S.M. (2010). Breast cancer in young women. Breast Cancer Res..

[9] Bergquist J.R., Leiting J.L., Habermann E.B., Cleary S.P., Kendrick M.L., Smoot R.L., Nagorney D.M., Truty M.J., Grotz T.E. (2019). Early-onset gastric cancer is a distinct disease with worrisome trends and oncogenic features. Surgery.

[10] Bilimoria K.Y., Talamonti M.S., Tomlinson J.S., Stewart A.K., Winchester D.P., Ko C.Y., Bentrem D.J. (2008). Prognostic Score Predicting Survival After Resection of Pancreatic Neuroendocrine Tumors: Analysis of 3851 Patients. Ann. Surg..

[11] Han X., Xu X., Jin D., Wang D., Ji Y., Lou W. (2014). Clinicopathological characteristics and prognosis-related factors of resectable pancreatic neuroendocrine tumors: A retrospective study of 104 cases in a single Chinese center. Pancreas.

[12] Yang M., Tian B.L., Zhang Y., Su A.P., Yue P.J., Xu S., Wang L. (2014). Evaluation of the World Health Organization 2010 grading system in surgical outcome and prognosis of pancreatic neuroendocrine tumors. Pancreas.

[13] Liu J.B., Baker M.S. (2016). Surgical Management of Pancreatic Neuroendocrine Tumors. Surg. Clin. N. Am..

[14] Schurr P.G., Strate T., Rese K., Kaifi J.T., Reichelt U., Petri S., Kleinhans H., Yekebas E.F., Izbicki J.R. (2007). Aggressive surgery improves long-term survival in neuroendocrine pancreatic tumors: An institutional experience. Ann. Surg..

[15] Howe J.R., Merchant N.B., Conrad C., Keutgen X.M., Hallet J., Drebin J.A., Minter R.M., Lairmore T.C., Tseng J.F., Zeh H.J. (2020). The North American Neuroendocrine Tumor Society Consensus Paper on the Surgical Management of Pancreatic Neuroendocrine Tumors. Pancreas.

[16] Ehehalt F., Saeger H.D., Schmidt C.M., Grutzmann R. (2009). Neuroendocrine tumors of the pancreas. Oncologist.

[17] Zikusoka M.N., Kidd M., Eick G., Latich I., Modlin I.M. (2005). The molecular genetics of gastroenteropancreatic neuroendocrine tumors. Cancer.

[18] Pieterman C.R., Conemans E.B., Dreijerink K.M., de Laat J.M., Timmers H.T., Vriens M.R., Valk G.D. (2014). Thoracic and duodenopancreatic neuroendocrine tumors in multiple endocrine neoplasia type 1: Natural history and function of menin in tumorigenesis. Endocr. Relat. Cancer.

[19] Thakker R.V., Newey P.J., Walls G.V., Bilezikian J., Dralle H., Ebeling P.R., Melmed S., Sakurai A., Tonelli F., Brandi M.L. (2012). Clinical practice guidelines for multiple endocrine neoplasia type 1 (MEN1). J. Clin. Endocrinol. Metab..

[20] Akerstrom G., Hessman O., Hellman P., Skogseid B. (2005). Pancreatic tumours as part of the MEN-1 syndrome. Best Pract. Res. Clin. Gastroenterol..

[21] Dean P.G., van Heerden J.A., Farley D.R., Thompson G.B., Grant C.S., Harmsen W.S., Ilstrup D.M. (2000). Are patients with multiple endocrine neoplasia type I prone to premature death?. World J. Surg..

[22] Nell S., Borel Rinkes I.H.M., Verkooijen H.M., Bonsing B.A., van Eijck C.H., van Goor H., de Kleine R.H.J., Kazemier G., Nieveen van Dijkum E.J., Dejong C.H.C. (2018). Early and Late Complications After Surgery for MEN1-related Nonfunctioning Pancreatic Neuroendocrine Tumors. Ann. Surg..

[23] Binkovitz L., Johnson C.D., Stephens D. (1990). Islet cell tumors in von Hippel-Lindau disease: Increased prevalence and relationship to the multiple endocrine neoplasias. AJR. Am. J. Roentgenol..

[24] Libutti S.K., Choyke P.L., Bartlett D.L., Vargas H., Walther M., Lubensky I., Glenn G., Linehan W.M., Alexander H.R. (1998). Pancreatic neuroendocrine tumors associated with von Hippel Lindau disease: Diagnostic and management recommendations. Surgery.

[25] Larson A., Hedgire S., Deshpande V., Stemmer-Rachamimov A., Harisinghani M., Ferrone C., Shah U., Thiele E. (2012). Pancreatic neuroendocrine tumors in patients with tuberous sclerosis complex. Clin. Genet..

[26] Luo G., Javed A., Strosberg J.R., Jin K., Zhang Y., Liu C., Xu J., Soares K., Weiss M.J., Zheng L. (2017). Modified staging classification for pancreatic neuroendocrine tumors on the basis of the American Joint Committee on Cancer and European Neuroendocrine Tumor Society Systems. J. Clin. Oncol..

[27] Zhou H., Zhang Y., Wei X., Yang K., Tan W., Qiu Z., Li S., Chen Q., Song Y., Gao S. (2017). Racial disparities in pancreatic neuroendocrine tumors survival: A SEER study. Cancer Med..

[28] Yao J.C., Hassan M., Phan A., Dagohoy C., Leary C., Mares J.E., Abdalla E.K., Fleming J.B., Vauthey J.-N., Rashid A. (2008). One hundred years after “carcinoid”: Epidemiology of and prognostic factors for neuroendocrine tumors in 35,825 cases in the United States. J. Clin. Oncol..

[29] Sanford N.N., Aguilera T.A., Folkert M.R., Ahn C., Mahal B.A., Zeh H., Beg M.S., Mansour J., Sher D.J. (2019). Sociodemographic Disparities in the Receipt of Adjuvant Chemotherapy Among Patients With Resected Stage I–III Pancreatic Adenocarcinoma. J. Natl. Compr. Cancer Netw..

[30] Ordonez J.E., Hester C.A., Zhu H., Augustine M., Porembka M.R., Wang S.C., Yopp A.C., Mansour J.C., Zeh H.J., Polanco P.M. (2020). Clinicopathologic Features and Outcomes of Early-Onset Pancreatic Adenocarcinoma in the United States. Ann. Surg. Oncol..

[31] Raimondi S., Maisonneuve P., Löhr J.-M., Lowenfels A.B. (2007). Early onset pancreatic cancer: Evidence of a major role for smoking and genetic factors. Cancer Epidemiol. Prev. Biomark..

[32] Piciucchi M., Capurso G., Valente R., Larghi A., Archibugi L., Signoretti M., Stigliano S., Zerboni G., Barucca V., La Torre M. (2015). Early onset pancreatic cancer: Risk factors, presentation and outcome. Pancreatology.

[33] Massarweh N.N., Chiang Y.-J., Xing Y., Chang G.J., Haynes A.B., You Y.N., Feig B.W., Cormier J.N. (2014). Association between travel distance and metastatic disease at diagnosis among patients with colon cancer. J. Clin. Oncol..

[34] Youngwirth L.M., Nussbaum D.P., Thomas S., Adam M.A., Blazer III D.G., Roman S.A., Sosa J.A. (2017). Nationwide trends and outcomes associated with neoadjuvant therapy in pancreatic cancer: An analysis of 18 243 patients. J. Surg. Oncol..

[35] Deyo R.A., Cherkin D.C., Ciol M.A. (1992). Adapting a clinical comorbidity index for use with ICD-9-CM administrative databases. J. Clin. Epidemiol..

[36] Consortium A.P.G. (2017). AACR Project GENIE: Powering precision medicine through an international consortium. Cancer Discov..

[37] Ho D.E., Imai K., King G., Stuart E.A. (2011). MatchIt: Nonparametric Preprocessing for Parametric Causal Inference. J. Stat. Softw..

[38] Austin P.C. (2011). An introduction to propensity score methods for reducing the effects of confounding in observational studies. Multivar. Behav. Res..

